# When Less Is More: Non-monotonic Spike Sequence Processing in Neurons

**DOI:** 10.1371/journal.pcbi.1004002

**Published:** 2015-02-03

**Authors:** Hinrich Arnoldt, Shuwen Chang, Sven Jahnke, Birk Urmersbach, Holger Taschenberger, Marc Timme

**Affiliations:** 1 Network Dynamics, Max Planck Institute for Dynamics and Self-Organization (MPIDS), Göttingen, Germany; 2 Institute for Nonlinear Dynamics, Faculty of Physics, Georg August University Göttingen, Göttingen, Germany; 3 Department of Membrane Biophysics, Max Planck Institute for Biophysical Chemistry, Göttingen, Germany; 4 Bernstein Center for Computational Neuroscience (BCCN) Göttingen, Göttingen, Germany; Université Paris Descartes, Centre National de la Recherche Scientifique, FRANCE

## Abstract

Fundamental response properties of neurons centrally underly the computational capabilities of both individual nerve cells and neural networks. Most studies on neuronal input-output relations have focused on continuous-time inputs such as constant or noisy sinusoidal currents. Yet, most neurons communicate via exchanging action potentials (spikes) at discrete times. Here, we systematically analyze the stationary spiking response to regular spiking inputs and reveal that it is generically non-monotonic. Our theoretical analysis shows that the underlying mechanism relies solely on a combination of the discrete nature of the communication by spikes, the capability of locking output to input spikes and limited resources required for spike processing. Numerical simulations of mathematically idealized and biophysically detailed models, as well as neurophysiological experiments confirm and illustrate our theoretical predictions.

## Author Summary

Brain function relies on robust communication between a huge number of nerve cells (neurons) that exchange short-lasting electrical pulses (called action potentials or spikes) at certain times. How nerve cells process their spiking inputs to generate spiking outputs thus is key not only to individual neurons’ computational capabilities but also to the collective dynamics of neuronal networks. Here we analyze the response properties of neurons to regular spike sequence inputs. We find that neurons typically respond in a non-monotonic way. Output frequency mostly increases with input frequency as expected but sometimes output frequency necessarily decreases upon increasing the input frequency. Our theoretical analysis predicts that spiking neurons commonly exhibit such non-monotonic response properties. Simulations of simple mathematical and complex computational models as well as neurophysiological experiments confirm our theoretical predictions.

## Introduction

Most neurons in the nervous system communicate by sending and receiving stereotyped electrical pulses called action potentials or spikes [1]. The computational capabilities of neural circuits centrally rely on the input-output relations of single neurons. This relation is commonly characterized by its output spike rate in response to a temporally continuous constant input current *I*, sometimes in the presence of additional current fluctuations [2–5].

If the spikes each neuron receives are irregular in time and individually only weakly affect the neuron’s membrane potential (the main physical quantity characterizing its dynamical state), this continuous-input picture serves as an appropriate approximation [6–8] to the actual spike sequence input. A neuron’s response curve in terms of its output spike rate as a function of input current is thus considered one of its most fundamental standard characteristics [5, 9–11]. In particular, neurons are dynamically classified according to such response curves (so-called *f*-*I*-curves), into type-I neurons, with their output spike rates increasing from zero above a critical current *I*_*c*_, and type-II neurons which start spiking with a macroscopic, non-zero rate upon increasing *I* beyond some *I*_*c*_ [10, 11]. For both types, neuronal output spike frequencies depend monotonically on the input *I*.

Yet, a broad range of neural systems, including most central pattern generators, pacemaker cells and neural pathways in the auditory and motor systems, exhibit more regular, patterned spike sequences [12–28] such that the above mean field approximation does not apply. Some experimental and numerical studies [29, 30] hint that certain neurons receiving regular periodic spiking inputs may exhibit locked spike responses together with possibly non-monotonic input-output relations. How common this phenomenon is and which neural features may cause non-monotonic response curves, however, remains unknown.

Here, joining theoretical, computational and experimental approaches we provide a systematic analysis of the response of individual neurons to regular spiking inputs. We find that non-monotonic response is a universal feature that emerges due to a locking phenomenon [31] in a broad range of systems. We reveal the underlying mechanisms and conclude that an arbitrary system (neuronal or otherwise pulse-coupled) will inevitably show such non-monotonic response, if it exhibits pulsed outputs locked to the pulsed inputs and any form of resource limitation during pulse processing.

The article is structured as follows: we first derive analytical results on idealized neuron models; based on insights from this model, we identify general theoretical (sufficient) conditions underlying non-monotonicity. Second, we observe that these conditions commonly occur across neural systems and check their robustness against temporal jitter and their occurrence in biophysically more detailed models. Third, neurophysiological experiments confirm and illustrate our finding for real neurons.

## Results

### Non-monotonic spike sequence responses abound

**Figure 1:**
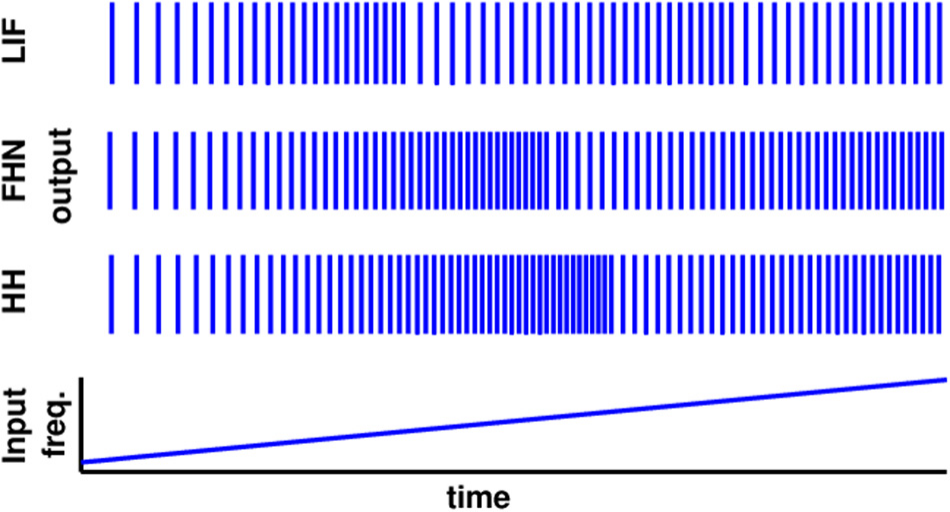
Non-monotonic response to regular input spike sequences: increasing the input spike frequency may increase but also decrease the output spike frequency. Bottom panel: input spike frequency that slowly increases ten-fold. Top three panels: output spike responses (LIF: leaky integrate-and-fire neuron with depressive synapse, FHN: Fitzhugh-Nagumo and HH: Hodgkin-Huxley neuron, both with static synapses). Time is rescaled so that all three data sets fit in this Figure. For details of models see equations (1)–(2) for LIF, equations (12)–(13) for FHN and equations (14)–(17) for HH.

Studying first the response properties of leaky integrate-and-fire (LIF) neurons [1] receiving periodic spiking input sequences via depressive synapses [32] (cf. Fig. 1), we find that slowly increasing the input frequency initially also increases the output spike frequency (as expected). When crossing certain input frequencies, however, the output frequency drops again, implying a non-monotonic response. Our extensive numerical studies show that this phenomenon robustly emerges for various combinations of neural time scales, synaptic time scales, synaptic efficacies and other features of the system. Checking various other types of neuron models including Fitzhugh-Nagumo and Hodgkin-Huxley neurons, this phenomenon persists, even with static synapses. Fig. 1 illustrates three examples. These findings led us to hypothesize that non-monotonic responses to regular spike sequence inputs are a generic feature across neurons. To test this hypothesis, we first answered the question what causes such non-monotonic response.

### Theoretical analysis

To reveal the basic ingredients underlying non-monotonic response, we analyzed an idealized, mathematically tractable system consisting of a LIF neuron receiving spiking input via a depressive synapse, i.e. a synapse that is weakened on short time scales when transmitting a pulse. The LIF-model [1] is a standard model for analyzing the dynamics of spiking neural systems and a standard model for depressive synapses was introduced by Tsodyks and Markram [32]. In their simplest setting, the combined dynamics of voltage *V*(*t*) and synaptic resources *x*(*t*) are given by

$$\dot{V}\;=\;\frac{V_{\text{eq}}-V}{\tau }+cx\sum_{m=-\infty }^{\infty }\delta (t-t_{m}),$$ (1)

$$\dot{x}\;=\;\frac{1-x}{\mu }-ux\sum_{m=-\infty }^{\infty }\delta (t-t_{m}).$$ (2)

Here *τ* is the membrane time constant, *V*_eq_ defines the equilibrium potential, the constant *c* ∈ ℝ is the maximum possible response of the neuron to one incoming spike, it is modulated by the amount *x*(*t*) of resources available at time *t*; further, *μ* is the time constant of resource recovery, *u* is the fraction of available resources depleted per spike and *t*_*m*_ defines the time of the *m*th spike received by the neuron. Upon crossing a threshold *V*(*t*^−^) ≥ *V*_Θ_ ≔ 1, the neuron emits a spike and the membrane potential is reset, *V*(*t*) ≔ *V*_reset_ ≔ 0. With *V*_eq_ < 1 the neuron is an excitable system and may only send a spike at times it receives an input spike.

What is the stationary spike response of this system to regular spiking input sequences with inter-spike intervals of fixed length *T*? Analytically integrating the coupled system (1)–(2) between two subsequent input spike times, we derive an event map

$$\left(\begin{array}{c} V(t_{m+1})\\ x(t_{m+1}) \end{array}\right)=\left(\begin{array}{c} \left[V\left(t_{m}\right)-V_{\text{eq}}\right]e^{-T/\tau }+V_{\mathrm{eq}}+cx\left(t_{m+1}\right)\\ \left[x\left(t_{m}\right)\left(1-u\right)-1\right]e^{-T/\mu }+1 \end{array}\right)$$ (3)

from its state at input spike time *t*_*m*_ to its state at the next input spike time *t*_*m*+1_ = *t*_*m*_ +*T*. Here, *V*(*t*_*m*_) denotes the membrane potential directly *after* the receiption of the spike and *x*(*t*_*m*_) denotes the state of the synapse directly *before* the transmission of the spike. The resource dynamics of this map depend only on the input interval *T*, but are independent of the potential dynamics. Thus, we directly find the fixed point of the resource dynamics defined by x^*^(*t*_*m*+1_) = x^*^(*t*_*m*_). As the response map is linearly dependent on *x* with slope ∣(1−*u*)*e*^−*T*/*μ*^∣ < 1 the fixed point is globally stable. A simple analysis reveals the fixed point

$$x^{*}\left[T\right]=\frac{1-\exp(-T/\mu )}{1-(1-u)\exp(-T/\mu )}$$ (4)

to which the resource dynamics converges exponentially from all initial conditions. Assuming stationarity, we derive the dynamics of the membrane potential starting just after reset at (*V*(*t*_0_), *x*(*t*_0_)) ≔ (0,x^*^) and consider the subthreshold membrane potential dynamics as a function of the number *n* ≥ 1 of incoming spikes. Note that from this initial condition at all input spike times, we have *x*(*t*_*m*_) = x^*^ by construction.

Iterating the membrane potential dynamics in the event map (3) *n* times from the initial condition (*V*(*t*_0_), *x*(*t*_0_)) = (0, x^*^) yields

$$\begin{array}{ccc} V(t_{n}) & = & cx^{*}\left[T\right]\sum_{m=1}^{n}e^{-(n-m)T/\tau }+V_{\mathrm{eq}}\left(1-e^{-nT/\tau }\right)\;. \end{array}$$ (5)

which we prove by induction.

*Induction basis:* For *n* = 1, (5) becomes

$$\begin{array}{ccc} V(t_{1}) & = & cx^{*}\left[T\right]+V_{\mathrm{eq}}\left(1-e^{-T/\tau }\right) \end{array}$$ (6)

which is the first iteration of the potential dynamics in (3) for *V*(*t*_0_) = 0, *x*(*t*_0_) = x^*^ and *t*_1_ − *t*_0_ = *T*.

*Induction step:* Assume (5) holds for some *n* ∈ ℕ. Substituting (5) into (3) we obtain

$$\begin{array}{ccc} V(t_{n+1}) & = & \left(V\left(t_{n}\right)-V_{\mathrm{eq}}\right)e^{-T/\tau }+V_{\mathrm{eq}}+cx^{*}\left[T\right]\\ & = & \left(cx^{*}\left[T\right]\sum_{m=1}^{n}e^{-(n-m)T/\tau }+V_{\mathrm{eq}}\left(1-e^{-nT/\tau }\right)-V_{\mathrm{eq}}\right)e^{-T/\tau }+V_{\mathrm{eq}}+cx^{*}\left[T\right]\\ & = & cx^{*}\left[T\right]\sum_{m=1}^{n}e^{-(n+1-m)T/\tau }+cx^{*}\left[T\right]+V_{\mathrm{eq}}\left(1-e^{-(n+1)T/\tau }\right)\\ & = & cx^{*}\left[T\right]\sum_{m=1}^{n+1}e^{-(n+1-m)T/\tau }+V_{\mathrm{eq}}\left(1-e^{-(n+1)T/\tau }\right) \end{array}$$ (7)

which is equation (5) with *t*_*n*_ = *nT* replaced by *t*_*n*+1_ = (*n*+1)*T*.

Evaluating the geometric sum, (5) further simplifies to

$$V\left(t_{n}\right)=\left(1-e^{-nT/\tau }\right)\left(\frac{cx^{*}\left[T\right]}{1-e^{-T/\tau }}+V_{\mathrm{eq}}\right)$$ (8)

which is the membrane potential immediately *after* the *n*th spiking input received after potential reset at *t*_0_.

As a consequence, the first time *t*_*ñ*_ where *V*(*t*_*ñ*_) ≥ 1 defines the number of input spikes *ñ* required for the neuron’s potential to reach threshold and thus for the neuron to emit a spike. For certain combinations of system parameters and input frequencies, where $\left(\frac{cx^{*}[T]}{1-e^{-T/\tau }}+V_{\text{eq}}\right)<1$, no positive integer *ñ* makes expression (8) larger than one. Thus, the neuron does not emit any spike and has output frequency *λ*_out_ = 0. For all other combinations, we find

$$\tilde{n}=\left\lceil -\frac{\tau }{T}\;\text{ln}\left(1-\frac{1}{V_{\text{eq}}+\frac{cx^{*}[T]}{1-e^{-T/\tau }}}\right)\right\rceil .$$ (9)

**Figure 2:**
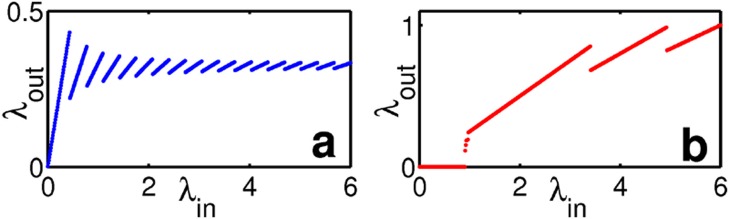
Non-monotonic response functions of the idealized LIF synapse-neuron system. Input-output response (a) for resource recovery that is much slower than the time scale of membrane potential leakage (*τ* = 1, *μ* = 10), for system with dynamics shown in Fig. 1, (b) for both processes occurring on the same time scales (*τ* = 1, *μ* = 1). In (a) only downward jumps, in (b) upward as well as downward jumps occur. Further parameters were *u* = 0.2, *c* = 0.5, *V*_eq_ = 0.8 for (a) and *u* = 0.4, *c* = 0.8, *V*_eq_ = 0 for (b).

Here we denote by ⌈*z*⌉ the smallest integer larger than *z*. As the input frequency of a periodic spike sequence is given by *λ*_in_ = 1/*T* and the output period by $T_{\text{out}}=\tilde{n}T=\lambda_{\text{out}}^{-1}$, combining (9) with (4) yields

$$\lambda_{\text{out}}=\lambda_{\text{in}}\times {\left\lceil -\tau \lambda_{\text{in}}\;\text{ln}\left(1-\frac{1}{V_{\text{eq}}+\frac{c\frac{1-\text{exp}(-1∕(\mu \lambda_{\text{in}})}{1-(1-u)\text{exp}(-1∕(\mu \lambda_{\text{in}})}}{1-\text{exp}(-1∕(\tau \lambda_{\text{in}}))}}\right)\right\rceil }^{-1}$$ (10)

as the output frequency of this neuron-synapse system. This input-output relation exhibits qualitatively different shapes depending on system parameters. For instance, the neuron will spike for arbitrarily low input frequencies for one set of parameters while at others it only spikes when receiving inputs beyond a certain minimal frequency, see Fig. 2 for an illustration.

### Locking and limited resources suffice

What do relations (9)–(10) tell us about the spiking response of the neuron? First, there are intervals where the output frequency is locked to the input frequency by an integer *ñ*. This locking ratio in turn changes in a jump-like manner at certain critical *λ*_in_. Thus, in the neuron’s response curve, the output frequency *λ*_out_ either increases proportionally to the input frequency *λ*_in_ (with constant slope 1/*ñ*), or – at the critical frequencies – *ñ* changes by one and the response changes qualitatively to a different proportional dependence. Interestingly, depending on the time scales in the system and the coupling constants, *ñ* may not only decrease, but also increase by increasing of *λ*_in_, thereby lowering the response frequency by increasing the input. Fig. 2 shows two qualitatively different examples. Indeed, as our prior numerical results suggested, an increase in *ñ* and thus a downward jump in the response curve, implying its non-monotonicity, is a persistent feature in this simple neuron-synapse system.

This non-monotonic response does not rely on dynamic features of the synapse: Whereas the exact form of (10) is specific to the simple synapse-neuron system considered above, the main mechanism underlying non-monotonic input-output relations is rooted in the fact that in systems with periodic pulsed inputs and outputs locking *per se* occurs only between an integer number of pulses. One key observation is that a formula similar to (10) is (at least) implicitly defined across spiking neuron models, specifying the output spike frequency *λ*_out_ as a function of input frequency *λ*_in_ in the regime of locking. Such an expression would be of the form

$$\lambda_{\text{out}}=\lambda_{\text{in}}\times {\left\lceil f(p;\lambda_{\text{in}})\right\rceil }^{-1}$$ (11)

where *f*(p;*λ*_in_) is some function of all model parameters **p** and the input frequency. As for the simple LIF system above, continuously changing the input frequency *λ*_in_ implies an output frequency *λ*_out_ proportional to *λ*_in_ within certain intervals where *f*(**p**;*λ*_in_) has real values between two integers. At frequencies *λ*_in_ where *f*(**p**;*λ*_in_) ∈ ℕ exactly equals an integer, the response *λ*_out_ jumps. Non-monotonicities emerging this way may be shielded for instance by noise, measurement errors or intrinsic obstacles to locking in complex neurons.

There are thus three conditions sufficient for this phenomenon to emerge: (i) the discrete nature of communication by pulses (such as spikes), (ii) an output pulse sequence that locks to the input sequence and (iii) any type of resource limitation in pulse-transmission.

As a consequence, it is not important where the resources are expended, whether in the presynaptic part, during intinsic processing in the postsynaptic neuron, or during action potential initiation in that neuron: in particular, non-monotonicity will also occur in neurons without dynamic synapses, as soon as any resources (e.g. ions, vesicles, neurotransmitters, calcium, or others) are needed for the spike transmission. The limited resources may thus either be expended in the presynaptic synaptic terminal leading to depressive synaptic transmissions, or may consist of the configuration of the membrane proteins which cannot change state twice within the relevant time scales. Moreover, the amount of charge required for action potential generation is limited and may be viewed as limited resources in the general theoretical sense. Finally, the notion of resources also relates to the fact that neurons exhibit a so-called refractoriness, so that immediately after the emission of a spike a neuron cannot emit a second spike due to a lack of resources.

### More complex neuron models

**Figure 3:**
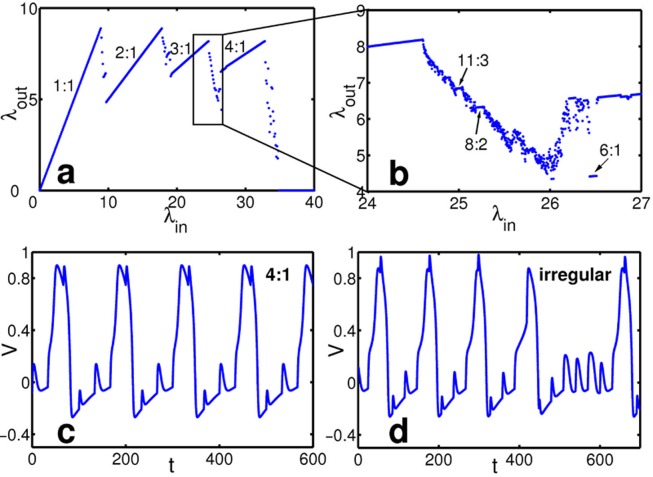
Non-monotonic response in a Fitzhugh-Nagumo neuron receiving periodic input via a static synapse. (a) Input-output response exhibits dominant *n:1*-locking interrupted by broad transition regions (b), magnified from (a). Several locking ratios *n:m* are indicated. In the transition regions, periodic, *n:m*-locked as well as nonperiodic, irregular dynamics arise. (c,d) Membrane potential dynamics (c) in the *4:1*-locking region and (d) in the irregular regime. The model parameters were *a* = 0.139, *b* = 2.54, *c* = 0.5, *μ*′ = 125 and *K*(*t*) = 2(exp(−t)−exp(−2*t*)).

Thus, the nonmonotonic response properties should persist in more complex neuron models and real biological neurons. We first numerically demonstrate this in two further example classes of neuronal model systems. We start with a Fitzhugh-Nagumo neuron [33] with temporally extended postsynaptic currents receiving spiking input via a static synapse. This model is defined by the membrane potential *V*(*t*) and a gating variable *W*(*t*). The differential equations

$$\dot{V}\;=\;-V(V-1)(V-a)-W+c\sum_{m=-\infty }^{\infty }K(t-t_{m})$$ (12)

$$\dot{W}\;=\;\frac{V-bW}{\mu^{\prime }}$$ (13)

define their time evolution where *a* is a parameter setting the equilibrium membrane potential, *b* defines the opening rate of ion channels, *c* is the input strength arriving via a static synapse and *μ*′ is related to the gating time constant. *K*(*t*) is the kernel modeling the post-synaptic response to incoming spikes. The second output spike train in Fig. 1 demonstrates that the output rate can decrease with increasing input frequency in this neuron model. We studied this model systematically by recording the mean output frequency for different input frequencies in simulations. These simulations again reveal a non-monotonic input-output relation (Fig. 3a), albeit a more complicated one than for the more abstract model discussed above: As before, wherever the input is locked to the output, the response frequency necessarily increases in proportion to the input frequency. Between such bands of simple *n:1-*locking, we now observe extended transition regions exhibiting either more complex locking (such as, e.g., *11:3*) with periodic output as well as unlocked, aperiodic output spike sequences (Fig. 3b-d). We attribute such broader transition regions to the higher-dimensional membrane potential dynamics that, together with the temporally extended spike response (and possibly numerical noise) includes a mechanism for the generation of spikes of different amplitudes and thus makes the system amenable of “skipping” a full spike (cf. Fig. 3c,d).

**Figure 4:**
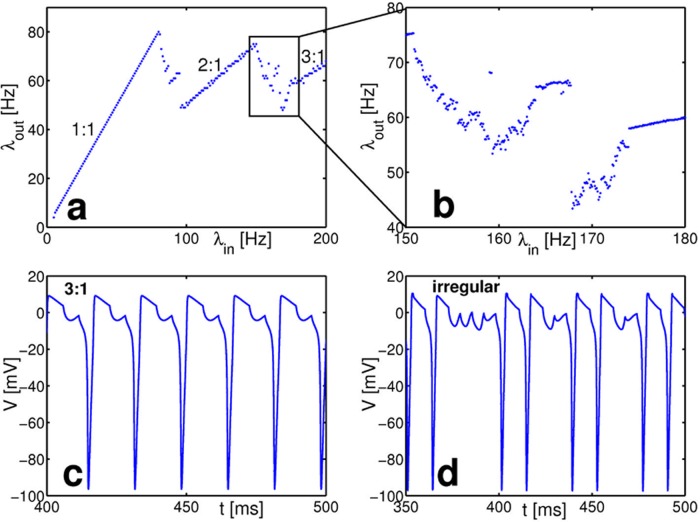
Non-monotonic response in a Hodgkin-Huxley neuron receiving periodic input via a static excitatory synapse. (a) In the response curve *n:1*-locking regions are interrupted by broad transition regions (b), magnified from (a). In the transition regions nonperiodic, irregular dynamics arise. (c) and (d) show example dynamics of the membrane potential (c) in the *3:1*-locking region (*λ*_in_ = 170Hz) and (d) in the irregular regime (*λ*_in_ = 140.2Hz). Simulation parameters were *C* = 2, *V*_Na_ = 50, *V*_K_ = −77, *V*_L_ = −54.4, *g*_Na_ = 120, *g*_K_ = 36, *g*_L_ = 0.3, *I*_0_ = 5 and *ε* = 9. We used an alpha-function $K(t)=e\frac{t}{\tau_{\text{ex}}}\exp\left(-t/\tau_{\text{ex}}\right)$ with time constant *τ*_ex_ = 1 to model the synaptic inputs (*e* is the Euler constant to normalize *K*(*t*)).

The biophysically more detailed Hodgkin-Huxley model yields qualitatively the same results, in particular non-monotonic input-output relations (cf. also third spike train in Fig. 1). As an example, Fig. 4 illustrates the input-output relation of a standard Hodgkin-Huxley neuron where we followed [5, 34] to describe the neuron by four dynamic variables

$$\dot{V}\;=\;\frac{1}{C}\left[I_{\text{ex}}+I_{0}-g_{\text{Na}}m^{3}h(V-V_{\text{Na}})-g_{\text{K}}n^{4}(V-V_{\text{K}})-g_{\text{L}}(V-V_{\text{L}})\right]$$ (14)

$$\dot{m}\;=\;\left(\frac{0.1(V+40)}{1-e^{-(V+40)/10}}\right)\left(1-m\right)-4e^{-(V+65)/18}m$$ (15)

$$\dot{n}\;=\;\left(\frac{0.01(V+55)}{1-e^{-(V+55)/10}}\right)(1-n)-0.125e^{-(V+65)/80}n$$ (16)

$$\dot{h}\;=\;0.07e^{-(V+65)/20}(1-h)-\frac{1}{1+e^{-(V+35)/10}}h,$$ (17)

where *I*_ex_ is the input current arriving via an excitatory synapse

$$I_{\text{ex}}=\epsilon \sum_{m}K(t-t_{m}).$$ (18)

In the above set of equations, *V* is the membrane potential in mV, *m*, *n*, and *h* are dimensionless gating variables, *I*_ex_ is the input current in *μ*A/cm^2^ arriving via an excitatory synapse and *I*_0_ is a constant input current in *μ*A/cm^2^. *C* is the membrane’s capacity in *μ*F/cm^2^, *g*_Na_, *g*_K_ and *g*_L_ are the maximal specific conductances for sodium (Na) and potassium (K) induced currents and the leakage current through the membrane (L) in mS/cm^2^.*V*_Na_, *V*_K_ and *V*_L_ denote the corresponding equilibrium (reversal) potentials in mV. *K*(⋅) is the dimensionless kernel function defining the shape of the synaptic response and *ε* specifies the strength (peak amplitude) of single inputs in *μ*A/cm^2^. Time *t* and reception times *t*_*m*_ are given in ms.

In direct numerical simulations of this complex four-dimensional neuron model, we find input-output relations that are qualitatively similar to the responses described above. Fig. 4 shows that Hodgkin-Huxley neurons exhibit locked as well as irregular dynamics. The input-output relation increases for frequencies with locked dynamics and decreases in the transition regions with irregular dynamics, akin to those in the simpler Fitzhugh-Nagumo model. We conclude that this behaviour persists even in complex high-dimensional neuron models.

### Insensitivity to jitter in the input spike times

Does the non-monotonicity of the response curve depend on the exact periodicity of the inputs? To answer this question we simulated the dynamics of the above described LIF model system with the input pulse intervals Δ*t* being Gamma-distributed, i.e.

$$p(\Delta t)=\frac{\lambda^{\alpha }e^{-\lambda \Delta t}\Delta t^{\alpha -1}}{\Gamma (\alpha )}$$ (19)

where *λ* is the scale parameter and *α* is the shape parameter. The mean input interval is then $\bar{\Delta t}=\alpha /\lambda$, so that the input rate is defined by *λ*_in_ = *λ*/*α*. The relative standard deviation of the input intervals is $\sigma_{\Delta t}/\bar{\Delta t}=1/\sqrt{\alpha }$.

**Figure 5:**
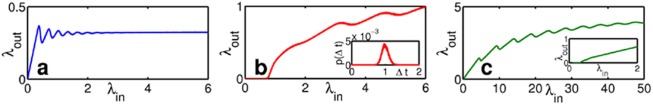
Non-monotonicity of response curves is robust against irregularity of the input. Panels (a)–(c) show the input-output response of the LIF model system receiving input spike sequences with Gamma-distributed inter-spike intervals. The system parameters in (a) and (b) are identical to the system parameters in Fig. 2(a),(b). (c) shows the response of the system for parameters *τ* = 4, *μ* = 1, *u* = 0.2, *c* = 0.5 and *V*_eq_ = 0, where the inset demonstrates that for small input rates no output is generated. Inset of (b) shows the distribution for one fixed input rate, normalized to one. The shape parameter of the distribution was set to *α* = 100, so that the relative standard deviation of the input intervals is $\sigma_{\Delta t}/\bar{\Delta t}=0.1$.

We again find a non-monotonic response function (see Fig. 5). Now, the infinitely fast downwards jumps (found for the deterministic input) become finite downward slopes due to the stochasticity of the input. In particular, we generally find that the non-monotonicity prevails. In the presence of any sufficiently small stochasticity the transition regions become more narrow and the frequency-decreasing jumps more pronounced the more regular the input sequences are. Based also on the theoretical finding that the main mechanism is rooted in generic resource limitation, we conclude that the phenomenon is also insensitive to fluctuations in input spike times. Only for highly irregular input the effect becomes indetectable as the dynamics are no longer locked to the input and the responses are dispersed due to the stochastic component in the input. In particular, the neuron’s response function is monotonic in the limit of uncorrelated (Poisson) spike inputs. This holds for systems without any resource limitation as well as for systems with intrinsic resource limitation [39–41]. For instance, spike frequency adaptation, one specific form of resource limitation, may turn a neuron into a high pass filter with a response function that is modified compared to neurons without adaptation but still monotonic [40, 41].

We expect similarly blurred non-monotonicity for other forms of stochastic inputs modifying regular periodic input spike trains, as discussed above. For instance, spike sequence inputs with missing (skipped) spikes or sufficiently correlated spike timings may induce related non-monotonic responses. For correlated inputs this has been, e.g., observed in [37, 38].

### Neurophysiological experiments: Biological neurons

The above theory predicts generic non-monotonic responses to regular spike sequences for all neurons that exhibit at least two locking regimes. Our simulations further indicate that non-monotonic responses persist in less perfect setups, and should thus also exist in real biological neurons. Here various types of noise and structural heterogeneities influence the neuron dynamics, specifically the exact timing of the membrane potential’s response to inputs and the spike generation mechanism act as additional stochastic componentes dispersing the output spikes in time. To check if the phenomenon persists despite this dispersal due to heterogeneities, we studied neuronal responses also experimentally. For our experiments we chose neurons from the medial nucleus of the trapezoid body (MNTB) of rats. Such MNTB neurons play an essential role in the auditory pathway of rodents [35] where they support to encode and transmit features of sound stimuli. They process spike sequences of much higher regularity than those typical for basic cortical circuits [26] with input frequencies ranging from a few to several hundreds of Hertz. For this relevant frequency range we stimulated such neurons *in vitro* with 1ms rectangular current pulses to emulate spiking inputs (see Methods section for details of slice preparation, electrophysiology and the analysis of recorded spike sequences).

**Figure 6:**
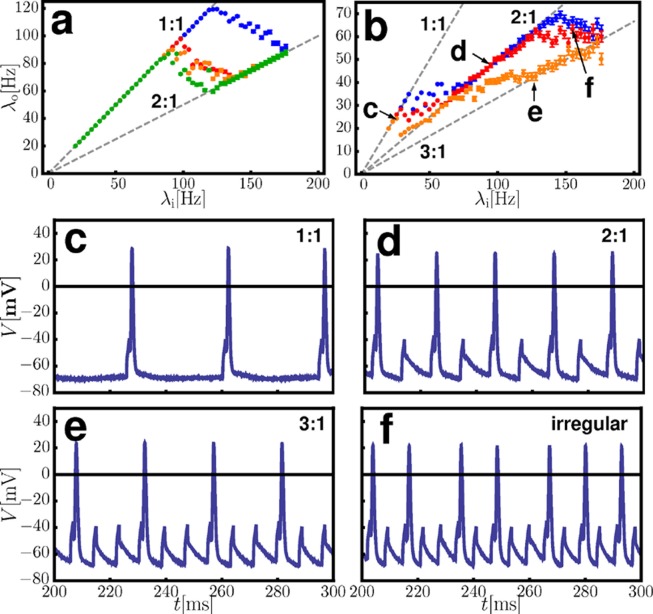
Non-monotonic response to regular inputs as observed in MNTB-neurons from whole-cell patch-clamp recordings. (a,b) Experimentally obtained response curves of two different MNTB-neurons for different pulse currents [(a) green: 375pA, orange, red: 425pA, blue: 450pA (b) orange: 575pA, red: 600pA, blue: 650pA]. The dashed lines indicate the major *n:1*-locking states predicted —no free fit parameter. Error bars indicate the estimated error made by calculating the mean output frequency from a finite number of output spikes. (c–f) Membrane potential dynamics for different locking types: (c) *1:1*-locking, (d) *2:1*-locking, (e) *3:1*-locking, (f) unlocked, irregular dynamics. The letters in (b) indicate the data points where these dynamics were observed.

Whole-cell patch-clamp recordings of MNTB neurons yielded non-monotonic response curves consistent with our theoretical predictions (Fig. 6). Panels (a) and (b) of Fig. 6 show the non-monotonic response curves for two different neurons, at three different stimulation amplitudes each. The recorded response of one (Fig. 6a) is apparently much less noisy than that of another (Fig. 6b). The overall picture is similar to the dynamics of the FHN-neurons studied above, with the exception that we did exclusively find irregular, unlocked dynamics in the transition regions, but no *n:m*-locking. We speculate that states of higher order *n:m*-locking are not sufficiently robust to be visible under such noisy conditions in inhomogeneous environments. Fig. 6(c-f) illustrates the membrane potential dynamics for the observed *1:1*-, *1:2*- and *1:3*-locking as well as for an example of unlocked, irregular response.

## Discussion

The above results provide a characterization of how regular spiking inputs to neurons yield spiking output responses with universal, system-independent features. This subject was broadly neglected so far as the focus was on responses to continuous-time inputs [24, 36, 42, 43]. That neuronal dynamics can lock to the input it receives is long known and the consequence of the locked dynamics and associated changes in the neuron’s response under certain conditions were observed before in specific neuronal systems [29, 30, 44–46]. Furthermore, previous studies found non-monotonic neuronal response to periodic spiking inputs [29, 30] and non-monotonic gain curves for neurons receiving correlated inputs via depressive synapses [37, 38]. However, the ultimate cause for the emerging non-monotonicity as well as its generality across systems’ details were not revealed in these studies. Our results now demonstrated that locking in systems that receive spiking inputs and exhibit any type of limited resource generically induces responses that are non-monotonic. We emphasize that this phenomenon relies on system-independent features and therefore is not restricted to the four systems (LIF, FHN, HH, MNTB neurons) studied here theoretically, computationally and experimentally. The above theoretical considerations demonstrate that any neuronal system exhibiting output locked to the spiking input generically exhibits non-monotonic response curves whenever resources are limited. Perfectly periodic inputs are not necessary for non-monotonic responses, but correlated inputs [37, 38] suffice to excite locking in the system and thus ultimately cause non-monotonic gain curves. This finding is backed by our simulations considering jitter in the input spike times. Whereas initially revealed for systems with adaptive synapses, it is also not relevant whether resources are mainly limited in the pre-synapse or during input processing within the postsynaptic cell itself, causing, e.g., refractoriness or other effective inhibition of an action potential.

How a neuron processes regular spike sequences thus serves as a general characteristic that is complementary to its *f*-*I*-response to continuous input currents [10, 11].

Interestingly, certain neurons may exhibit a different type of non-monotonic, typically unimodal response [42, 43]. Such responses, however, are markedly distinct from the phenomenon revealed above, because potential non-monotonicities in the output rate arise as a consequence of a subthreshold resonance due to a combination of noisy and oscillating continuous-time inputs and as such are unrelated to the direct locking of discrete time spikes.

Neurons exhibiting dynamical responses similar to those illustrated above (cf. Fig. 2a) on a coarse scale display a linear transmission function with saturation threshold such that any input substantially above the threshold (about *λ*_in_ ≈ 0.3 in our LIF example) would result in an output around that value, *λ*_out_ ≈ 0.3, thus providing a nonlinear computational element that roughly fixates the output if the frequency of the input sequence is sufficiently high.

More generally, non-monotonic single-neuron responses will have strong functional impacts already in simple neural circuits using spike-time codes [47–49]. As an example, even a short chain, small network motifs [50] and cyclic networks of a few neurons may reliably set an operating point and classify external inputs according to their frequency. We speculate that such basic computations may be essential in neural circuits that require stabilized spike sequences and as such be of central relevance for controlling behavior. Examples for such systems operating with close-to periodic spike sequences are central pattern generators — essential for controlling locomotion — and the CA1 area of the hippocampus exhibiting oscillations with a precise and stable frequency [12–22]. In these neural networks the nonmonotonic response properties of the single neurons may well be important for the stability of the overall network dynamics. This points towards the open question how the non-monotonic relations between spiking inputs and spiking outputs of single neurons impact the computational capabilities of neural circuits in general.

## Methods

The neurophysiological experiments on principal neurons of the medial nucleus of the trapezoid body (MNTB) were carried out in the following way.

### Slice preparation

Brainstem slices were prepared from Wistar rats (postnatal day 8 to 10) essentially as described before in [51]: the brainstem was quickly immersed in ice-cold low Ca^2+^ artificial cerebral spinal fluid (aCSF) containing (in mM): NaCl (125), KCl (2.5), MgCl_2_ (3), CaCl_2_ (0.1), glucose (25), NaHCO_3_ (25), NaH_2_PO_4_ (1.25), ascorbic acid (0.4), myo-inositol (3), Na-pyruvate (2), pH = 7.3 when bubbled with carbogen (95% O_2_, 5% CO_2_). The tissue was glued onto the stage of a VT1000S vibratome (Leica, Nussloch, Germany) and 200 µm thick slices were cut. Slices were transferred to an incubation chamber containing normal aCSF and maintained at 35°C for 30–40 min, and thereafter kept at room temperature (22–24°C) for at most 4 hours. The composition of normal aCSF was identical to low Ca^2+^ aCSF except that 1.0 mM MgCl_2_ and 2.0 mM CaCl_2_ were used.

### Electrophysiology

Whole-cell patch-clamp recordings were obtained from MNTB-neurons using an EPC-10 amplifier. The analog signals were digitized and stored on disk using ‘Pulse’ software (HEKA Elektronik, Lambrecht/Pfalz, Germany). Sampling intervals and filter settings were ≤20 µs and 4.5 kHz, respectively. All offline analysis was performed with ‘Igor Pro’ software (Wavemetrics, USA). Cells were visualized by IR-DIC microscopy through a 40x water-immersion objective (NA = 0.8) using an upright BX51WI microscope (Olympus, Germany) equipped with a 1.5–2x pre-magnification and a VX45 CCD camera (PCO, Germany). All experiments were carried out at room temperature. Patch pipettes were pulled from borosilicate glass with filament (Science Products GmbH, Hofheim, Germany) on a P-97 micropipette puller (Sutter Instrument, Novato, CA). Open tip resistance was 2–4 MΩ and access resistance (RS) was ≤20 MΩ. Action potentials (AP) trains were elicited by injecting trains of 50 short (1 ms duration) rectangular, depolarizing current pulses at various frequencies (20 to 176 Hz) and amplitudes (250 to 900 pA). The sweep interval was 200ms to allow voltage-activated conductances to fully recover from inactivation. APs were measured in the current-clamp mode of the EPC-10 after adjusting the fast-capacitance cancellation while in cell-attached mode. Pipette were filled with a solution consisting of (in mM): K-gluconate (100), KCl (60), HEPES (10), EGTA (5), Na2-phosphocreatine (5), ATP-Mg (4), GTP (0.3), pH = 7.3 with KOH.

### Analysis of recorded spike sequences

We extracted the output frequency from each data trace resulting from the measurements using a self-written program. This program counted the number of output spikes *N* generated by the MNTB neuron and registered the time *T* between the first and the last spike. Here, we made use of the fact, that the first input spike always generated an output spike. Then, the output frequency was given by *λ*_out_ = (*N*−1)/*T*. As we cut off the remaining data trace, where no further output spike was measured, we made a small error *e*. We estimated the size of this error in the following way: Whenever the input is locked *1:1* to the output, there is no error as we cannot miss any output spike interval. The probability of missing one interval is proportional to the difference of input and output frequency. As we measured the neuron’s output to 50 input spikes the error we make in missing one interval is 1/50^th^ of the overall measured frequency. Thus, we estimated the error to be *e* = (*λ*_in_−*λ*_out_)/50.

### Analysis of simulated spike sequences

As for recorded spike sequences, we counted the number *N* of generated spikes for a time interval of length at least Δ*t* ≥ Δt^*^ = 10*s* or 100 spikes, implying a maximum error of the output frequency of ∣Δ*f*∣ ≤ (*f*Δ*t*)^−1^ or ∣Δ*f*∣/*f* ≤ 1% resp.
